# Giant Modulation of Microstructure and Ferroelectric/Piezoelectric Responses in Pb(Zr,Ti)O_3_ Ultrathin Films via Single-Pulse Femtosecond Laser

**DOI:** 10.3390/nano15181450

**Published:** 2025-09-20

**Authors:** Bin Wang, Mingchen Du, Hu Wang, Mengmeng Wang, Dawei Li

**Affiliations:** 1 School of Optoelectronic Engineering and Instrumentation Science, Dalian University of Technology, Dalian 116024, China; 2 Laser Micro/Nano Fabrication Laboratory, School of Mechanical Engineering, Beijing Institute of Technology, Beijing 100081, China

**Keywords:** ferroelectric oxide thin films, single-pulse femtosecond laser, microstructural engineering, fs laser peening, ferroelectric and piezoelectric responses

## Abstract

Ferroelectric oxides, such as Pb(Zr,Ti)O_3_ (PZT), have been shown to maintain stable ferroelectricity even in ultrathin film configurations. However, achieving controllable modulation of microstructure and physical responses in these ultrathin films remains challenging, limiting their potential applications in modern nanoelectronics and optoelectronics. Here, we propose a single-pulse femtosecond (fs) laser micromachining technique for high-precision engineering of microstructure and ferroelectric/piezoelectric responses in ultrathin PZT films. The results show that various microstructures can be selectively fabricated through precise control of fs laser fluence. Specifically, nano-concave arrays are formed via low-fluence laser irradiation, which is mainly attributed to the fs laser peening effect. In contrast, nano-volcano (nano-cave) structures are generated when the laser fluence is close to or reaches the ablation threshold. Additionally, applying an fs laser pulse with fluence exceeding a critical threshold enables the formation of nano-cave structures with controlled depth and width in PZT/Pt/SiO_2_ multilayers. Piezoresponse force microscopy measurements demonstrate that the laser peening process significantly enhances the piezoelectric response while exerting minimal influence on the coercive field of PZT thin films. This improvement is attributed to the enhanced electromechanical energy transfer and concentrated compressive stresses distribution in PZT thin films resulting from the laser peening effect. Our study not only offers an effective strategy for microstructure and property engineering in ferroelectric materials at the nanoscale but also provides new insights into the underlying mechanism of ultrafast laser processing in ferroelectric thin films.

## 1. Introduction

In recent years, the rapid development of intelligent electronics and internet of things technologies has driven an increasingly urgent demand for high-speed and highly reliable memory devices in embedded systems [1,2]. Ferroelectric random access memory (FeRAM), with its non-volatility, low power consumption, and high read/write speed, has emerged as a promising candidate for next-generation memory applications [3,4]. Among FeRAM configurations, ferroelectric oxides [e.g., Pb(Zr,Ti)O_3_] and their heterostructures have attracted extensive attention due to their high storage density and tunable interfacial coupling effects [5,6,7]. Moreover, it has been shown that ferroelectric oxides can maintain stable ferroelectricity in ultrathin films even down to one-nanometer limit [8]. However, the modulation of microstructural and physical properties in ultrathin ferroelectric oxide films remains not fully explored [9,10,11,12], thereby limiting their potential applications in modern nanoelectronics and optoelectronics [13,14,15].

Femtosecond (fs) lasers, as ultrafast and ultra-intense energy carriers, enable high-precision micro-processing and induce phase changes in target materials within extremely short timescales [16,17,18,19]. Compared to longer laser pulses, fs lasers offer the key advantage of minimizing the heat-affected zone, which facilitates precise control of the microstructure features [20,21,22,23]. Previous studies have demonstrated that focused fs laser beams with fluence close to the ablation threshold can directly create craters, nano-bumps, jet-like structures, and complete material removal in various thin films [24,25,26,27]. Additionally, fs lasers have been widely applied in the fabrication of novel functional materials and the modulation of device performance [16,28,29]. However, the dynamic microstructural and physical responses of ferroelectric oxide ultrathin films under fs laser pulse irradiation remain largely unexplored.

In this work, we report on the high-precision engineering of microstructure and ferroelectric/piezoelectric responses in Pb(Zr,Ti)O_3_ (PZT) ultrathin films using a single-pulse fs laser micromachining technique. Optical microscopy and atomic force microscopy (AFM) characterizations reveal a progressive evolution of microstructures with increasing fs laser pulse fluence. When employing laser fluence below 0.5 nJ, nano-concave arrays are achieved in PZT thin films, which are attributed to the fs laser peening effect. As the laser fluence increases to 1 nJ, nano-volcano structures emerge, while ablation occurs at a fluence of approximately 2 nJ. Additionally, single-point fs laser irradiation with fluence exceeding 2 nJ enables the formation of nano-cave structures with controlled depth and width in PZT/Pt/SiO_2_ multilayers, resulting from a combination of fs laser ablation and melting effects. Piezoresponse force microscopy (PFM) measurements demonstrate that the laser peening process significantly enhances the piezoelectric response while exerting minimal influence on the coercive field of PZT ultrathin films, which is attributed to the enhanced electromechanical energy transfer and compressive stresses distribution in PZT thin films with nano-concave structures induced by laser peening effect. Our study provides an ultrafast, non-contact, laser-based approach for precisely modulating the microstructure and physical properties of ferroelectric ultrathin films, offering new pathways for the development of next-generation high-performance non-volatile memory devices.

## 2. Results and Discussion

**Figure 1:**
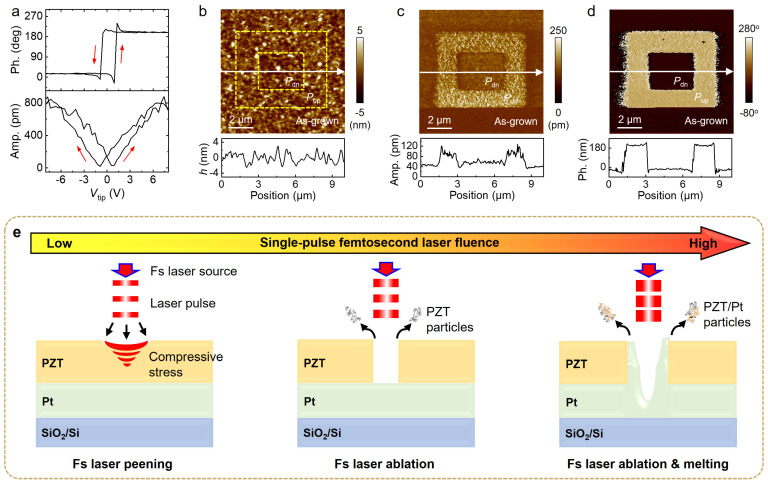
Characterizations of ferroelectric PZT thin films grown on a Pt/SiO_2_/Si substrate. (**a**) Switching hysteresis of (top) phase and (bottom) amplitude responses taken on as-grown PZT thin film (~50 nm). The red arrows illustrate the switching direction; (**b**) AFM topography, PFM (**c**) amplitude, and (**d**) phase images of square domains written on a PZT thin film. The lower panels in (**b**–**d**) show the cross-sectional signal profiles along the white solid lines. The yellow dashed lines in (**b**) mark the edges of PZT domain pattern. The dark and bright regions in (**d**) represent *P*_dn_ and *P*_up_ states, respectively; (**e**) Schematic view showing different interactions between PZT layers and fs laser pulse by varying the laser fluence: (left panel) laser peening effect, (middle panel) laser ablation effect, and (right panel) laser ablation and melting effects.

For the ferroelectric oxide layer, we worked with 50 nm thick PZT thin film deposited on SiO_2_ (285 nm)/Si substrate (Figure S1 in Supplementary Materials), with a 150 nm thick Pt buffer layer serving as the bottom electrode. The PZT thin films were grown by magnetron sputtering, followed by rapid thermal annealing (see Methods) to improve the crystallinity of the perovskite phase. To analyze the intrinsic ferroelectric characteristics of PZT thin films, we performed PFM studies (see Methods). Figure 1a displays the switching hysteresis of phase and amplitude responses taken on the as-grown PZT thin films, where the coercive voltages for the polarization-down (*P*_dn_) and polarization-up (*P*_up_) states are 0.78 V and −0.93 V, respectively. Figure 1b shows the AFM image of the PZT thin film deposited on Pt/SiO_2_/Si substrate, which exhibits relatively smooth surface with a root mean square of ~1.4 nm. Figure 1c,d represent the PFM amplitude and phase images of the box-in-box domain structure written on the 50 nm PZT film by applying a DC bias voltage of ±10 V to the AFM tip, which exhibits uniform *P*_dn_ and *P*_up_ domain pattern. The above PFM characterizations confirm the exceptional polarization controllability and nanoscale domain engineering capability in the as-grown PZT ultrathin films.

Various laser-material interaction dynamics are considered to exist between single-pulse fs laser irradiation and PZT ultrathin films (Figure 1e). As shown in Figure 1e (left panel), during low-fluence single-pulse fs laser irradiation, high-energy photons induce a nonlinear ionization effect that liberates surface electrons from atomic confinement, resulting in the generation of transient high-density plasma. The rapid expansion of this plasma produces ultrahigh-pressure shock waves that significantly exceed the material’s yield strength, thereby inducing plastic deformation in the PZT layer [30,31]. This shock- peening effect is anticipated to enable surface modification of PZT thin films at nanoscale spatial resolution, such as the formation of nano-concave structures. When the single-pulse fs laser fluence approaches the critical threshold, ablation begins to occur, leading to the formation of nano-volcano (nano-cave) structures in the PZT thin film (Figure 1e (middle panel)). Further increasing fs laser fluence beyond this threshold simultaneously triggers ablation in the PZT layer and melting in the Pt layer (Figure 1e (right panel)), resulting in the formation of more complex nano-concave structures [32,33].

**Figure 2:**
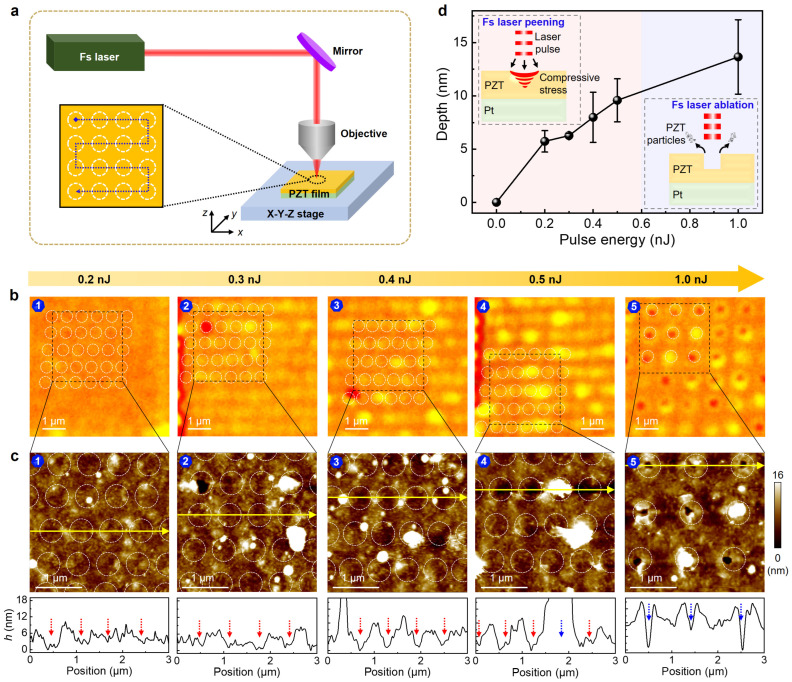
Fabrication of nano-concave arrays in PZT thin films by fs laser peening. (**a**) Schematic illustration of microstructure engineering in PZT thin films by single fs laser pulse scan (scanning pitch: ~0.96 μm); (**b**) Optical images and (**c**) AFM topographies of nanostructures generated on PZT thin films by single fs laser pulse irradiation at different laser fluences (ranging from 0.2 nJ to 1 nJ). The white dashed circles in (**b**,**c**) represent fs laser pulse irradiation sites. The lower panels in (**c**) show the cross-sectional height profiles along the yellow solid lines. The red dotted arrows point to the peening action area (0.2–0.5 nJ), while the blue arrows point to the ablation action area (0.5–1 nJ); (**d**) Dependence of nano-concave (or nano-volcano) depth, with error bars, on fs laser pulse fluence. The insets show schematics for nano-concave and nano-volcano formation mechanisms via a single fs laser pulse.

Based on the above assumption, we next employ a single-pulse fs laser micromachining technique for high-precision nano-engineering of PZT/Pt/SiO_2_ multilayers, through which various nanostructures can be controllably fabricated. Figure 2a schematically illustrates the fabrication of nanostructure arrays in the PZT thin films via single fs laser pulse scan. A fs laser beam with a wavelength of 1030 nm was focused onto the surfaces of the PZT thin films, with a high-precision electrically controlled sample stage enabling two-dimensional (2D) scanning for nanostructure array fabrication. The spacing of nanostructure array can be well controlled by adjusting the laser scanning speed. Figure 2b,c and Figure S2 show the optical and AFM images of PZT thin films by single fs laser pulse irradiation at different laser fluences (ranging from 0.2 nJ to 1 nJ) with a scanning pitch of approximately 0.96 μm. The white dashed circles represent fs laser pulse irradiation sites, and a distinct fluence-dependent nanostructure evolution is observed. When employing a weak laser fluence of 0.2 nJ, no apparent structural change is optically resolvable (Figure 2(b1)). However, the corresponding AFM topography and cross-sectional profile analyses reveal periodic height modulation (Figure 2(c1)), indicative of weak shock-peening effect induced by single-pulse fs laser irradiation [34]. The laser shock-peening effect has been predominantly applied to enhance fatigue resistance in bulk and metallic materials such as stainless steel [35]. Here, we for the first time, realize fs laser peening–induced nanostructure engineering in ferroelectric oxide ultrathin films.

Upon increasing the single-pulse fs laser fluence to 0.3 nJ, discernible surface modification emerges optically (Figure 2(b2)), while AFM topography shows that the depth of depression intensifies (Figure 2(c2)), confirming the enhancement of laser-peening effect. Remarkably, under 0.4–0.5 nJ single-pulse fs laser irradiation conditions, the shock-peening effect becomes more pronounced (Figure 2(b3,b4)), which leads to the formation of relatively uniform and distinct nano-concave arrays in PZT thin films (Figure 2(c3,c4)). It should be noted that few particulate-like protrusions are observed in nano-concave arrays with the same laser power applied, which can be explained by fs laser power fluctuation during the scanning process (see Section 4). In contrast, under 1 nJ single-pulse fs laser irradiation, nano-volcano arrays are formed in the PZT thin film (Figure 2(b5,c5)), which can be attributed to thermomechanical coupling effects involving radial migration and resolidification of molten material [36]. Hence, 1 nJ is the critical transition threshold of PZT thin film between the shock peening–dominated regime (<1 nJ) and ablation-initiated regime (≥1 nJ).

Figure 2d quantitatively analyzes the dependence of nanostructure (nano-concave or nano-volcano) depth on fs laser fluence. Here, the depth is defined as the height difference in the modified zone between adjacent peak level and valley level. In the weak fluence region (0.2–0.5 nJ), the depth of nanostructures (nano-concaves) linearly increases, reflecting a highly fluence-dependent nonthermal shock-peening effect (left inset). With a relatively high fluence (~1 nJ), nano-volcano structures are formed and show significant depth fluctuation, suggesting that thermal ablation begins to occur beyond this threshold (right inset). The established fluence-dependent nanostructure evolution rules provide critical guidance for single-pulse fs laser high-precision nano-engineering in ferroelectric ultrathin films.

**Figure 3:**
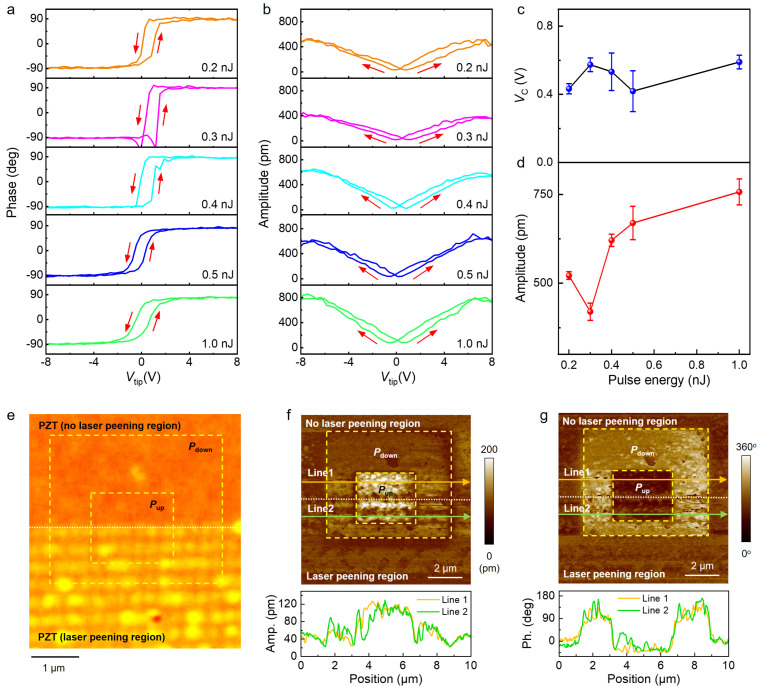
Effect of fs laser peening on the ferroelectric/piezoelectric properties of PZT thin films. Switching hysteresis of (**a**) PFM phase and (**b**) PFM amplitude responses taken from PZT nanostructures fabricated with fs laser peening at different laser pulse fluences (from top to bottom: 0.2 nJ, 0.3 nJ, 0.4 nJ, 0.5 nJ, and 1 nJ). The red arrows illustrate the switching direction; The dependence of (**c**) coercive voltage and (**d**) maximum amplitude with error bars on fs laser pulse fluence; (**e**) Optical image of a selected region in a PZT thin film, with the upper half showing no fs laser–peened area and the lower half showing fs laser–peened area. The white dotted line marks the boundary of these two areas; (**f**) PFM amplitude and (**g**) PFM phase images of the same region in (**e**), with square domains written in both fs laser-treated and untreated areas. The lower panels in (**f**,**g**) show the cross-sectional signal profiles along line 1 and line 2.

Next, we explore the effect of single-pulse fs laser irradiation on the ferroelectric and piezoelectric responses in PZT ultrathin films using the PFM technique. PFM has been proven to be a reliable approach that can be used to quantitatively characterize the ferroelectric and piezoelectric properties of various ferroelectric materials in local areas [37,38,39]. Figure 3a and Figure S3 compare the switching hysteresis of PFM phase response taken on PZT nanostructure arrays fabricated with fs laser peening at different laser fluences, where similar phase hysteresis curves are detected. Figure 3c quantitatively analyzes the dependence of coercive voltage (*V*_C_) extracted from Figure 3a and Figure S3 on fs laser fluence. It is clear that the average *V*_C_ remains stable (~0.5 ± 0.1 V) across a wide laser fluence range of 0.2 nJ to 1.0 nJ. This indicates that the ferroelectric polarization switching energy barrier does not alter. Thus, we can conclude that the fs laser-peening process exerts minimal influence on the ferroelectric properties of PZT ultrathin films. Figure 3b compares the switching hysteresis of PFM amplitude response taken from the same sample in Figure 3a at different laser fluences. The dependence of maximum amplitude extracted from Figure 3b and Figure S3 on fs laser fluence is shown in Figure 3d. It is found that, with increasing the laser fluence, the amplitude signal continuously increases, revealing significant enhancement of piezoelectric performance in PZT thin films.

The experimental results in Figure 2 have shown that nano-concave structures can be formed in PZT thin films by single-pulse fs laser irradiation, which is mainly attributed to the fs laser peening effect. Laser peening is a mechanical (cold working) process, where short pulses hit the surface of a PZT thin film and shockwaves are generated. These shockwaves can plastically deform the film surface and compressive residual stresses extend into the internal structure of PZT thin films [34,40]. Therefore, the piezoelectric enhancement in PZT thin films with nano-concaves can be attributed to fs laser-peening induced microstructure engineering. On the one hand, the formation of nano-concave structures enhances the electromechanical energy transfer, thus leading to an enhancement of the piezoelectric response [29]. On the other hand, the nano-concave structures enable a concentrated distribution of compressive stresses in the PZT thin films. The enhanced strain in the PZT thin films with nano-concave structures further promotes the enhancement of the piezoelectric response [29].

To further confirm the effect of laser peening on the ferroelectric/piezoelectric responses in PZT thin films, we performed ferroelectric domain pattern writing in both laser-peened and un-peened regions. Figure 3e shows the optical image of a PZT thin film partially processed with fs laser peening at a fluence of 0.5 nJ, where the upper half corresponds to the un-peened region, and the lower half represents the laser-peened region. Figure 3f,g shows the PFM amplitude and phase images of the square domain structure written on the same region as in Figure 3e by applying a bias voltage of ±10 V to the AFM tip. It is clear that both laser-peened and un-peened regions exhibit uniform *P*_dn_ and *P*_up_ domain pattern, thus confirming that fs laser peening does not suppress, but rather enhances the ferroelectric/piezoelectric responses of PZT thin films. The established fs laser fluence and property correlation offers new pathways for developing high-performance ferroelectric non-volatile memory devices.

The aforementioned discussion is mainly focused on the dynamic responses of PZT ultrathin films under fs laser irradiation at relatively low fluence levels (≤1 nJ), where complete ablation does not occur. In the following section, we investigate the impact of fs laser ablation on the microstructural characteristics of PZT thin films. It is predicted that complete ablation would occur at a fluence of 2 nJ, through which nano-cave structures could be obtained (Figure 1e (middle panel)).

**Figure 4:**
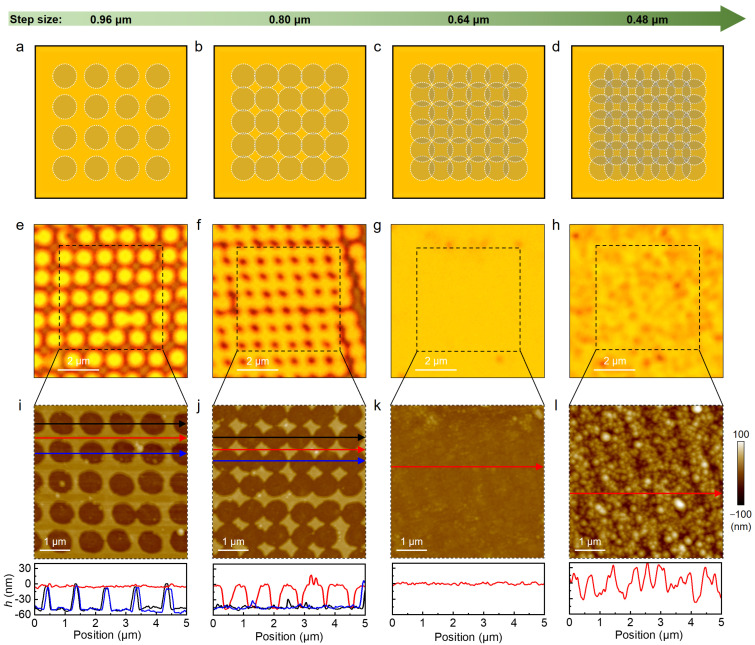
Fabrication of nano-cave arrays in PZT thin films by fs laser ablation. Schematics of nano-cave array formation in PZT thin film by fs laser scan at different step sizes: (**a**) 0.96 μm, (**b**) 0.80 μm, (**c**) 0.64 μm, and (**d**) 0.48 μm; Optical images of nano-cave array fabricated on PZT thin film with a fs laser pulse fluence of 2 nJ at different step sizes: (**e**) 0.96 μm, (**f**) 0.80 μm, (**g**) 0.64 μm, and (**h**) 0.48 μm. The bright and dark colors in (**e**,**f**) represent laser processed and unprocessed regions, respectively; AFM topographies of nano-cave array fabricated on PZT thin film (black boxes in (**e**–**h**)) with a fs laser pulse fluence of 2 nJ at different step sizes: (**i**) 0.96 μm, (**j**) 0.80 μm, (**k**) 0.64 μm, and (**l**) 0.48 μm. The lower panels in (**i**–**l**) show the cross-sectional height profiles along the solid lines. The black (blue) and red curves represent the height profiles along the center and edge positions of nano-cave arrays, respectively.

The scanning pitch plays an important role in the nanostructure array fabrication. In Figure 4a–d, we designed nanostructure arrays to be formed in PZT thin film by single-pulse fs laser scan at different step sizes (ranging from 0.96 to 0.48 μm). Figure 4e,i show the optical and AFM images of the nanostructure arrays fabricated in PZT thin film with a fluence of 2 nJ at a step size of 0.96 μm, confirming the achievement of uniform and periodic nano-cave structures. The corresponding high-magnified AFM image is shown in Figure S4. The cross-sectional height profiles reveal that the average depth of nano-cave structure is 50 nm, which is in good agreement with the thickness of the PZT thin film. It is demonstrated that single-pulse fs laser irradiation possesses the capability for atomic-level, selective removal of ultrathin films in the (PZT/Pt/SiO_2_) multilayer system.

When the step size is reduced to 0.8 μm (Figure 4b), as expected, nanoscale rhombic arrays are formed in the PZT layer, as evidenced by optical microscopy and AFM topography characterizations (Figure 4f,j). By reducing the step size to 0.64 μm (Figure 4c), we achieve the complete removal of the PZT layer (Figure 4g,k), without causing damage to the underlying Pt layer. This outcome can be attributed to the first-order overlap effect, which results in continuous spatial superposition of the fs laser beam. Concurrently, the accumulated equivalent pulse count within the overlapping region reaches approximately two pulses per unit area (Figure 4c). This value is sufficient to meet the ablation threshold of the PZT layer while remaining below the damage threshold of the Pt layer. When the step size is further reduced to 0.48 μm (Figure 4d), a secondary overlap effect occurs, increasing the pulse density to approximately three pulses per unit area. Under this condition, a remarkable increase in surface roughness is observed in the fs laser–scanned region (Figure 4h,l), indicating that the Pt layer has been damaged. The aforementioned discovery provides a universally applicable fs laser-based methodology for high-precision “etching” of ferroelectric ultrathin films, demonstrating substantial potential for the nano-fabrication of integrated ferroelectric memory devices and piezoelectric micro-electromechanical systems.

**Figure 5:**
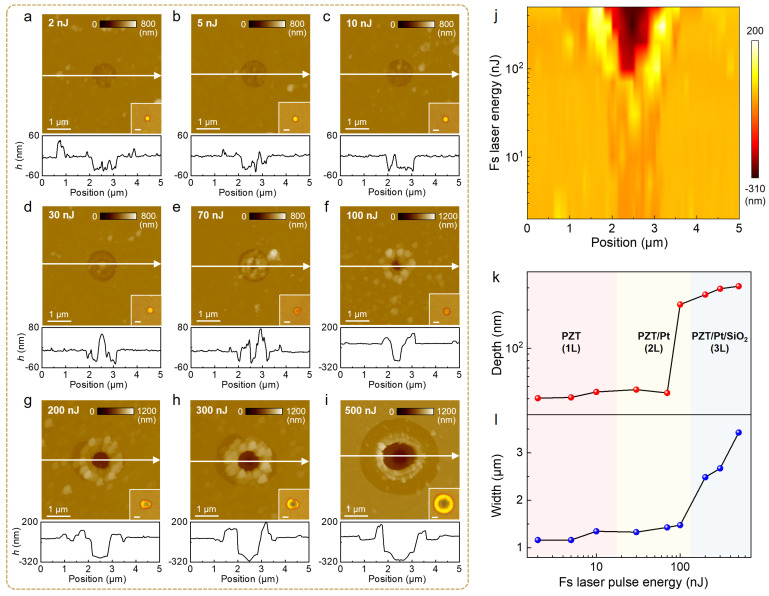
Nanostructure evolution in PZT/Pt/SiO_2_ multilayers as a product of laser pulse fluence under single-point fs laser irradiation. AFM topographies of nano-cave structures fabricated on PZT/Pt/SiO_2_ multilayers by single fs laser pulse with a laser fluence of (**a**) 2 nJ, (**b**) 5 nJ, (**c**) 10 nJ, (**d**) 30 nJ, (**e**) 70 nJ, (**f**) 100 nJ, (**g**) 200 nJ, (**h**) 300 nJ, and (**i**) 500 nJ. Insets: corresponding optical images; (**j**) 3D height profiles of nano-cave structures fabricated at different fs laser pulse fluences, extracted from the white lines in (**a**–**i**); (**k**) The dependence of nano-cave structure depth (red dots) on fs laser pulse energy; (**l**) The dependence of nano-cave structure width (blue dots) on fs laser pulse energy.

Finally, we investigate the controllable modulation of microstructures in PZT-based heterostructures under single-point fs laser irradiation. Figure 5a–i compare the AFM topographies of nanostructures fabricated on PZT/Pt/SiO_2_ multilayers using a single fs laser pulse with fluence ranging from 2 nJ to 500 nJ. The insets in Figure 5a–i show the corresponding optical images. In the relatively low fluence range (2 nJ to 10 nJ, Figure 5a–c), nano-cave structures are formed due to the laser ablation effect. These structures exhibit nearly consistent depth (~50 nm), while their diameter increases progressively with increasing fluence. At this stage, the applied fluence exceeds the ablation threshold of the PZT layer but remains below the damage threshold of the Pt/SiO_2_ substrate, enabling spatially selective removal of the PZT layer. In the medium-fluence range (30 nJ to 100 nJ), both AFM imaging and optical contrast (Figure 5d–f) indicate that fs laser melting and ablation occur in the Pt layer. Specifically, at a fluence of 30 nJ, a molten protrusion appears at the center of the laser-irradiated region (Figure 5d). This phenomenon is attributed to the Gaussian-type intensity profile of the laser beam [41], where only the central region receives sufficient fluence to exceed the melting point of the Pt layer. When the fluence increases to 70–100 nJ (Figure 5e,f), both ablation and melting processes occur in the Pt layer, resulting in the formation of nano-cavities at the central region. Concurrently, molten–solidified ring structures are observed in the peripheral area, which is attributed to the radial gradient of the laser energy distribution. In the high-fluence regime (200 nJ to 500 nJ), fs laser melting and ablation occur across the entire PZT/Pt/SiO_2_ multilayer. At 200 nJ, the initial modification of the SiO_2_ layer is observed. Upon increasing fluence to 300 nJ, a four-tier annular structure forms, consisting of an outermost PZT ablation zone, an intermediate Pt molten ring, an inner Pt ablation region, and a central SiO_2_ etching pit. At 500 nJ, this hierarchical architecture stabilizes and expands in both width and depth.

Figure 5j displays the 3D height profiles of nano-cave structures fabricated in PZT/Pt/SiO_2_ multilayer at different fs laser pulse fluences, offering a more intuitive understanding of the interaction between the fs laser and the multilayer system. In the lower energy regime, ablation of the PZT layer dominates the process, with the ablation diameter expanding as the laser fluence increases. When the fluence reaches a critical threshold, both the interaction depth and diameter increase abruptly. Figure 5k,i quantitively analyze the dependence of nano-cave structure depth and width on fs laser pulse fluence, respectively. It can be observed that below 100 nJ, both depth and width exhibit gradual increases, whereas above 100 nJ, exponential growth is evident. This indicates that the penetration depth and interaction area in the multilayer system do not scale linearly with fs laser fluence. By precisely adjusting the fs laser fluence, nano-cave structures with controlled depth and width can be fabricated in PZT/Pt/SiO_2_ multilayers.

## 3. Conclusions

In summary, we have realized giant modulation of microstructure, as well as ferroelectric and piezoelectric responses, in ultrathin (~50 nm) PZT films through single-pulse fs laser irradiation. By precisely controlling the fs laser fluence and scanning pitch, various nanostructures arrays, including nano-concave array, nano-volcano array, and nano-cave array, can be selectively fabricated within the PZT thin layer. It is found that the fs laser peening (ablation) effect primarily governs the formation of nano-concaves (nano-caves) in the PZT thin film, while a combination of fs laser ablation and melting effects enables the controlled fabrication of complex nano-cavities with tunable depth and width in the PZT/Pt/SiO_2_ multilayers. PFM investigations reveal that laser peening significantly enhances the piezoelectric performance while exerting minimal influence on the ferroelectric response of the PZT thin films. This enhancement is attributed to the increased electromechanical energy transfer and enhanced compressive stresses distribution in the nano-concaves induced by fs laser peening. These findings present an efficient and ultrafast laser-based strategy for precisely modulating the microstructure and functional properties of ferroelectric oxide thin films, opening new avenues for the development of high-performance non-volatile memory devices.

## 4. Methods

Preparation of PZT Thin Films: Ferroelectric PZT thin films were deposited by radio-frequency (RF) sputtering method (TRP450, SKY Technology Development Co., Ltd., Shenyang, China) on the Pt/Ti-coated SiO_2_/Si substrates, followed by rapid thermal annealing (OTF-1200X-4-RTP, Hefei Kejing Materials Technology Co., Ltd., Heifei, China). First, Pt/Ti/SiO_2_/Si substrates were prepared by sputtering 20 nm Ti and then 150 nm Pt onto SiO_2_ (285 nm)/Si substrate. Second, PZT thin films were deposited using RF sputtering with a PbZr_0.48_Ti_0.52_O_3_ target. The deposition was carried out at 200 °C, with a gas flow of 70 sccm Ar and 0.5 sccm O_2_, under a working pressure of 1 Pa. The power of PbZr_0.48_Ti_0.52_O_3_ target (ZhongNuo Advanced Material Technology Co., Ltd., Beijing, China) was fixed at 67 W. The thickness of PZT film was controlled through the sputtering time. Third, rapid thermal annealing treatment was performed. Specifically, the sample was put into a rapid thermal furnace in an O_2_ environment. The temperature was increased to 650 °C with a heating rate of 10.5 °C/s, kept at 650 °C for 8 min, and then cooled to room temperature naturally.

AFM and PFM Measurements: The morphologies and thicknesses of the PZT thin films were measured using an AFM system (MFP-3D Origin, Oxford Instruments, Oxford, UK) with an AFM tip (Micromesh HQ:NSC14, k = 5) working in AC mode. PFM measurements were carried out using the same AFM system with a Pt-coated AFM tip (Micromesh HQ:NSC18, k = 2.8) in DART mode. For domain writing, a DC bias of ±10 V was applied to the AFM tip and the PZT thin films were grounded. For PFM imaging, a small AC voltage was applied to the AFM tip. All PFM phase and amplitude hysteresis loop measurements were conducted using the same Pt-coated AFM tip and under the same experimental conditions (resonant peak of AFM tip: 460 kHz; scanning voltage range: −8 V to 8 V; scanning frequency: 0.2 Hz).

Fs Laser Processing: Fs laser processing was conducted using a laser system with a wavelength of 1030 nm and a pulse duration of 138 fs (YC-FL-20-2000-IR, Hangzhou Yacto Technology Co., Ltd., Hangzhou, China). The laser output was linearly polarized, its power was controlled using a half-wave plate and a Glan prism. To enable high-resolution processing, the laser beam was focused onto the sample using an objective lens (100× magnification, working distance: 1 mm, NA = 0.9), achieving a focused spot size of approximately 0.63 μm. The samples were mounted on an electrically controlled three-axis sample stage and processed in ambient air. The system allowed for precise control of scanning speed and trajectory for micromachining applications. Laser parameters, such as pulse energy, were optimized based on the material and processing conditions to ensure effective micromachining and patterning results. All laser experiments were performed at a repetition rate of 1 kHz. For single-pulse irradiation, a signal generator was used to deliver a synchronization signal to the laser, and the laser was triggered at the rising edge of the signal. For scanning experiments, the laser operated continuously at 1 kHz while the focal spot diameter was matched with the translational speed of the three-dimensional stage. The laser stability was characterized at a repetition rate of 100 kHz, where the average power was 0.5 mW and the long-term stability value was 0.3% over a continuous test of approximately 14 h. In addition, the root-mean-square power fluctuation was measured to be less than 0.7%.

## Data Availability

Dataset available on request from the authors.

## Supplementary Materials

The following supporting information can be downloaded at: https://www.mdpi.com/article/10.3390/nano15181450/s1, Figure S1: (a) AFM topography of the PZT thin film grown on Pt-coated SiO_2_/Si substrate. (b) The cross-sectional height profile along the white solid line in (a); Figure S2: (a) Low magnified AFM images of PZT nanostructures in Figure 2 in the main text with fs laser peening at laser pulse fluence ranging from 0.2 nJ to 1 nJ. (b) The corresponding high magnified AFM images in (a); Figure S3: Switching hysteresis of PFM phase and amplitude responses taken on PZT nanostructures at different regions fabricated with fs laser peening at laser pulse fluence of (a1–a3) 0.2 nJ, (b1–b3) 0.3 nJ, (c1–c3) 0.4 nJ, (d1–d3) 0.5 nJ, (e1–e3) 1 nJ; Figure S4: (a–d) Low magnified AFM images of PZT nanostructures in Figure 4 in the main text with fs laser peening by fs laser scan at step size of (a) 0.96 μm, (b) 0.80 μm, (c) 0.64 μm, and (d) 0.48 μm). (e–h) The corresponding high magnified AFM images in (a–d).
