# Supplementary material for: Giant Modulation of Microstructure and Ferroelectric/Piezoelectric Responses in Pb(Zr,Ti)O3 Ultrathin Films via Single-Pulse Femtosecond Laser
Source: Nanomaterials (Basel). 2025 Sep 20;15(18):1450. doi: 10.3390/nano15181450 (PMC12472458; doi:10.3390/nano15181450)
Supplement: Supplementary file 1 [file nanomaterials-15-01450-s001.zip › nanomaterials-3844541-supplementary.pdf]

nanomaterials

Supplementary Materials

Giant Modulation of Microstructure and Ferroelectric/Piezoelectric Responses in Pb(Zr,Ti)O_3 Ultrathin Films via Single-Pulse Femtosecond Laser

Bin Wang [1,†], Mingchen Du [2,†], Hu Wang [1], Mengmeng Wang [2] and Dawei Li [1,*]

1 School of Optoelectronic Engineering and Instrumentation Science, Dalian University of Technology, Dalian 116024, China
2 Laser Micro/Nano Fabrication Laboratory, School of Mechanical Engineering, Beijing Institute of Technology, Beijing 100081, China
* Correspondence: dwli@dlut.edu.cn
† These authors contributed equally to this work.

S1. Thickness measurement of PZT thin films

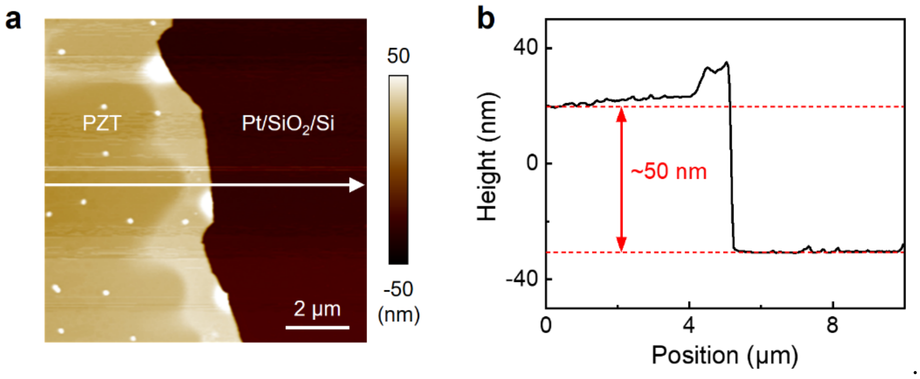

Figure S1. (**a**) AFM topography of the PZT thin film grown on Pt-coated SiO_2/Si substrate. (**b**) The cross-sectional height profile along the white solid line in (a).

S2. AFM images of PZT thin films by fs laser peening.

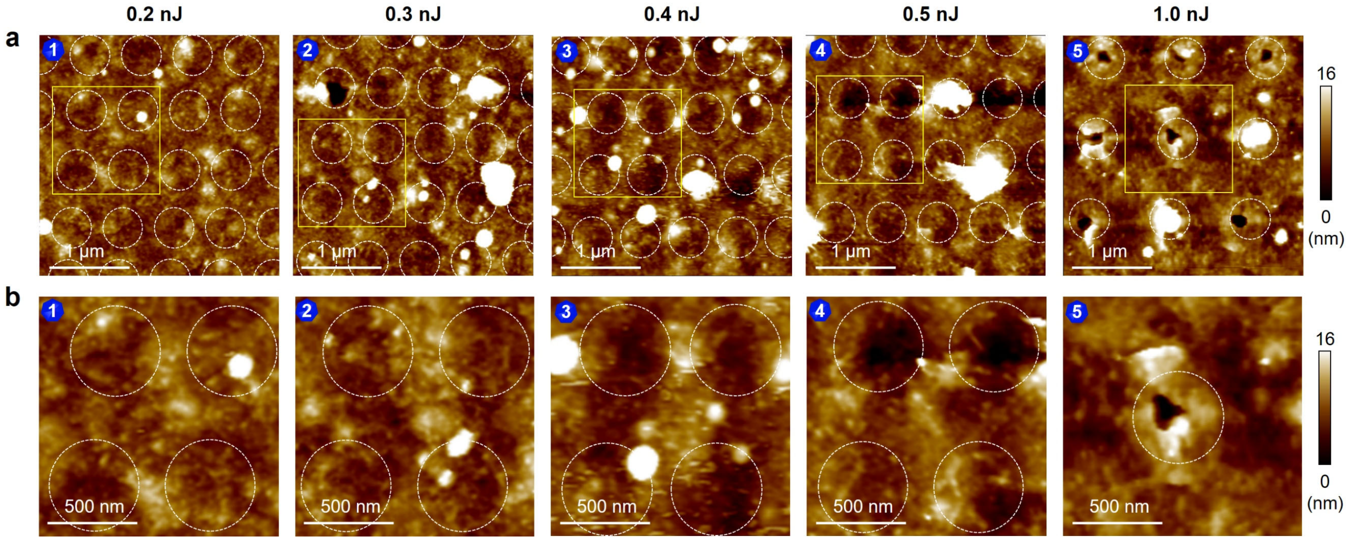

Figure S2. (**a**) Low magnified AFM images of PZT nanostructures in Figure 2 in the main text with fs laser peening at laser pulse fluence ranging from 0.2 nJ to 1 nJ; (**b**) The corresponding high magnified AFM images in (a).

S3. PFM measurement of PZT nanostructures

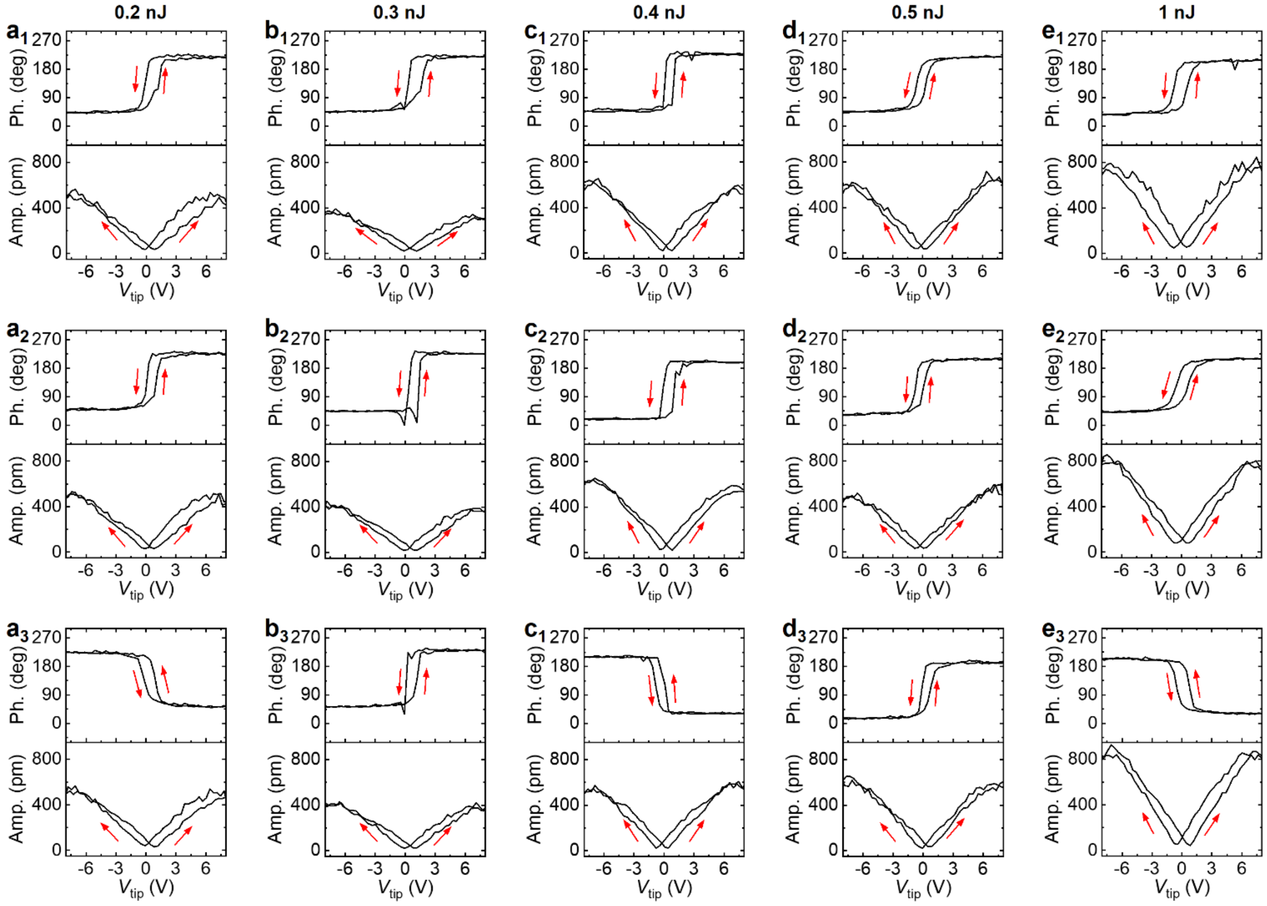

Figure S3. Switching hysteresis of PFM phase and amplitude responses taken on PZT nanostructures at different regions fabricated with fs laser peening at laser pulse fluence of (**a1–a3**) 0.2 nJ; (**b1–b3**) 0.3 nJ; (**c1-c3**) 0.4 nJ; (**d1–d3**) 0.5 nJ; (**e1–e3**) 1 nJ.

S4. AFM images of PZT thin films by fs laser ablation.

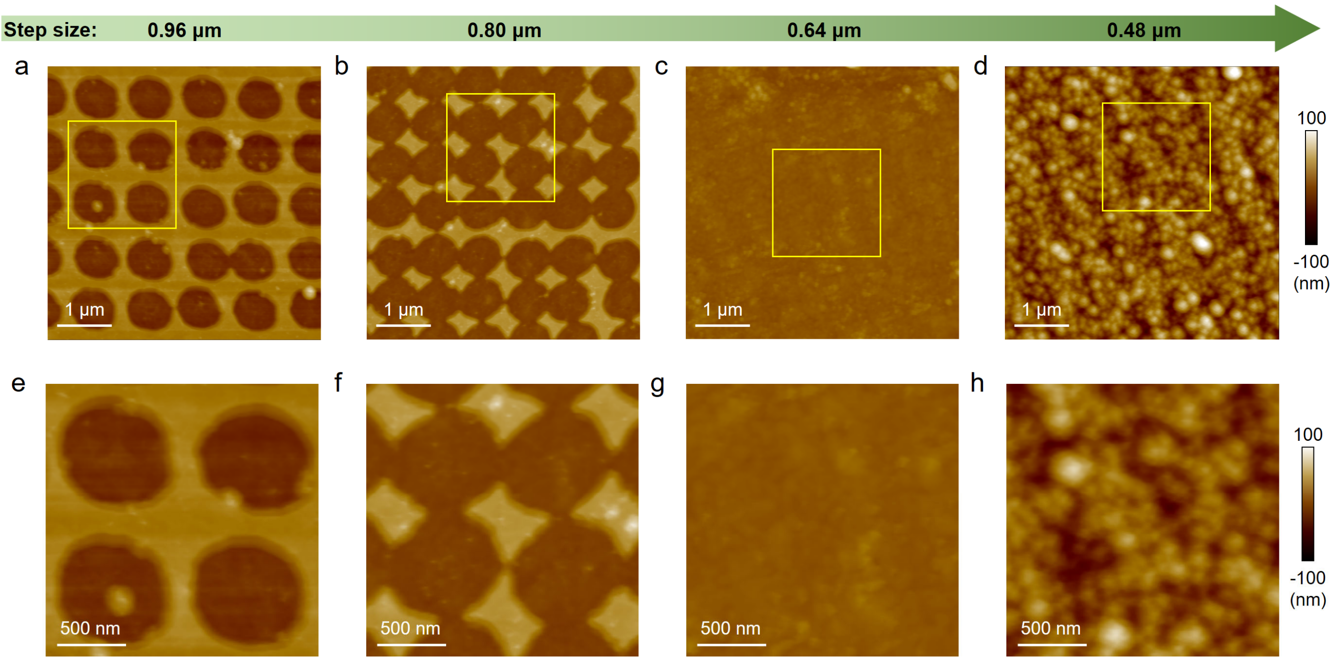

Figure S4. (**a–d**) Low magnified AFM images of PZT nanostructures in Figure 4 in the main text with fs laser peening by fs laser scan at step size of (a) 0.96 µm, (b) 0.80 µm, (c) 0.64 µm, and (d) 0.48 µm. (**e–h**) The corresponding high magnified AFM images in (a-d).
